# Attachment styles and maladaptive eating practices: perfectionism as a mediator

**DOI:** 10.1186/2050-2974-3-S1-P19

**Published:** 2015-11-23

**Authors:** Jai-Leigh Beard, Richard Hicks

**Affiliations:** 1 Bond University, Gold Coast, Australia

## 

The aims of this study were to investigate the relationship between attachment styles and maladaptive eating practices, and to determine whether the relationship was mediated by perfectionism. Attachment style, perfectionism and body image dissatisfaction have been examined separately in previous studies but the relationship with maladaptive eating practices has not been explored in community samples. The current study investigated whether perfectionistic tendencies mediated the relationship between attachment style and maladaptive eating practices. A total of 131 community individuals completed the following scales: Experiences in Close Relationships-Revised (ECR-R), Frost's Multidimensional Perfectionism Scale (FMPS), and the Maladaptive Eating Practices Questionnaire (MEPQ). Consistent with the research hypotheses, hierarchical regression established that maladaptive perfectionism mediated the relationship between attachment avoidance and maladaptive eating practices. In addition, maladaptive perfectionism mediated the relationship between attachment anxiety and maladaptive eating practices. Adaptive perfectionism also mediated the relationship between attachment anxiety and maladaptive eating practices but not between attachment avoidance and maladaptive eating practices. These findings suggest that perfectionistic tendencies whether ‘maladaptive’ or ‘adaptive’ act as an explanatory mechanism linking attachment style to maladaptive eating practices. Recommendations for professional practice and future research are suggested.

